# Global longitudinal strain can predict heart failure exacerbation in stable outpatients with ischemic left ventricular systolic dysfunction

**DOI:** 10.1371/journal.pone.0225829

**Published:** 2019-12-02

**Authors:** Damian Kaufmann, Małgorzata Szwoch, Joanna Kwiatkowska, Grzegorz Raczak, Ludmiła Daniłowicz-Szymanowicz

**Affiliations:** 1 Department of Cardiology and Electrotherapy, Medical University of Gdansk, Gdańsk, Poland; 2 Department of Pediatric Cardiology and Congenital Heart Defect, Medical University of Gdansk, Gdańsk, Poland; Universita degli Studi di Napoli Federico II, ITALY

## Abstract

**Background:**

Despite advancements in pharmacological and device-based treatment, heart failure (HF) continues to impose an enormous burden for health care system worldwide. Decompensation of HF is one of the main causes of hospitalization, therefore the identification of patients with the highest risk of such complication is still of great clinical importance. The prognostic significance and utility of global longitudinal strain (GLS) has been previously studied in patients with the broad spectrum of cardiovascular diseases in various endpoints, however its role in assessing the risk of hospitalization due to HF exacerbation of optimally treated outpatients has not been fully explored. Therefore, the aim of the study was to verify whether the GLS of the left ventricle (LV) derived by 2D speckle tracking echocardiography has, independently of other well-known clinical parameters, an additional impact on the risk of HF decompensation in stable patients with LV systolic dysfunction of ischemic origin.

**Methods:**

In 193 clinically stable HF outpatients with LV ejection fraction (LVEF) ≤ 50%, GLS, additionally to other clinical parameters, was analyzed. During 34 (14–71) months of follow-up, 58 patients were hospitalized due to HF decompensation (EVENT).

**Results:**

EVENT was significantly associated with age, QRS width, NYHA functional class, left atrium diameter, LV systolic and diastolic volume, LVEF, hemoglobin, brain natriuretic peptide, diuretic treatment, absence of beta-blockers, impaired renal function and history of diabetes in univariate Cox analyzes. GLS with pre-specified cut-off value of -9.4% was also significantly associated with the EVENT (HR 15.16; 95% CI 1.81–126.91). After adjusting for above-mentioned parameters GLS was still a significant predictor of hospitalization due to HF decompensation.

**Conclusions:**

GLS measurement can provide incremental information on the risk of HF decompensation in stable outpatients with LV systolic dysfunction of ischemic origin.

## Background

Constantly rising heart failure (HF) prevalence is a major clinical and public health concern. Despite compelling advancements in pharmacological and device-based treatment of HF, the mortality is still high [1, 2]. Coronary artery disease is a major risk factor for the incidence and decompensation of HF, which is the leading cause of hospitalizations, resulting in more than 1 million admissions in both the United States and Europe [3, 4]. From the clinical point of view, it seems crucial to identify the patients with the highest risk of such events and ensure that they receive expanded ambulatory care, which would translate into a reduced number of hospitalizations.

Over the years, many prognostic risk factors for HF exacerbation have been identified, including clinical, laboratory and echocardiographic parameters. Among the clinical parameters, the well-known are: new coronary events, poorly controlled hypertension, both ventricular and supraventricular arrhythmias with rapid ventricular rate, infections—especially respiratory system, high NYHA functional class, short distance in a 6-minute walk test, the presence of comorbidities, in particular: coronary artery disease, diabetes, renal failure, anemia and depression. In the group of laboratory parameters, increased levels of natriuretic peptides, troponins, catecholamine’s, urea, creatinine and low sodium levels have high prognostic value. In addition, one should not forget about other sociodemographic factors such as: age, marital status, compliance with dietary and therapeutic recommendations [5–9]. Many prior publications emphasize the usefulness ​​of left ventricular (LV) ejection fraction (LVEF) assessment, which was considered one of the most important risk factors for morbidity and mortality. Nevertheless better parameters are still sought. Two-dimensional speckle tracking echocardiography (2D STE) opens new diagnostic possibilities as a valuable tool to assess LV function. LV global longitudinal strain (GLS) has emerged as a parameter which is more sensitive and objective than LVEF in evaluation of LV abnormalities [10, 11]. This was firmly established in various studies as a reliable indicator of prognosis for the broad spectrum of cardiovascular diseases [12–19]. However, these studies were primarily focused on other endpoints than HF decompensation. Therefore, **the aim of our study** was to verify whether GLS of the LV derived by 2D STE provides, independently of other well-known clinical parameters, an additional impact on the risk of hospitalization due to HF decompensation in clinically stable outpatients with LV systolic dysfunction of ischemic origin.

## Methods

### Study design and patient population

Between October 2009 and October 2018 we prospectively enrolled consecutive stable patients with ischemic HF and LVEF ≤ 50%. The protocol of the study was approved by the Local Ethics Committee of the Medical University of Gdansk. Several clinical parameters were taken into account: a comprehensive baseline clinical history and physical examination, 12-lead ECG, routine laboratory blood tests and 2D-transthoracic echocardiography parameters, medical treatment and concomitant diseases. All patients had sinus rhythm and were clinically stable for at least 3 months before the enrollment. Patients received optimal medical therapy and coronary revascularization according to current guidelines [20–23]. The exclusion criteria were: age < 18 years, permanent atrial fibrillation/flutter, ventricular paced rhythm, NYHA functional class IV, clinical features of coronary instability at the moment of enrolment, a revascularization (coronary angioplasty or/and surgery by-pass) within 3 months prior to the study, or incomplete coronary revascularization status (scheduled control coronarography, coronary angioplasty or surgery by-pass), and non-cardiologic comorbidities with potential unfavorable effect on survival.

### Echocardiography

Transthoracic echocardiography was performed in patients in the left lateral decubitus position using a GE VIVID E9 ultrasound system (*GE Ultrasound*, *Horten*, *Norway*) equipped with phased-array transducer (M5S). Data were acquired from parasternal long-and short-axis views and the three standard apical views. For each view, cine loops were obtained by recording 3 consecutive heart cycles. Grayscale recordings were optimized for LV evaluation at a frame rate of 50–80 frames per second. All echocardiograms were stored digitally and analyzed with offline software on EchoPAC workstation (*v201*, *GE Healthcare*, *Horten*, *Norway*). Standard echocardiographic parameters such as: LV end-diastolic (EDV) and end-systolic (ESV) volume, left atrium diameter (LADs) were analyzed according to the principles described in the recommendations [24, 25]. LVEF was measured by Simpson’s biplane method.

#### Analysis of GLS

LV longitudinal function was assessed by the semiautomatic algorithm (*Automated Function Imaging*, *GE*, *Horten*, *Norway*). Briefly, three myocardial markers were placed in an end-diastolic frame in the apical 4-, 2- and 3-chamber views, respectively. The software automatically tracked the contour of the myocardium throughout the heart cycle to cover the entire thickness of the LV wall. Adequate tracking could be verified in real time and corrected by adjusting the region of interest or by manually correcting the contour to ensure optimal tracking. The value of peak GLS derived by 2D STE was analyzed from three apical views and calculated in the 17 segments (6 basal, 6 mid, 4 apical and the apex) in relation to the strain magnitude at aortic valve closure (identified on continuous wave Doppler recording through the aortic valve in 5-chamber view) [26]. The authors established that in the present study the higher absolute value of peak GLS (more negative) is assumed to be as better, and the value closer to zero is described as worse.

### Follow-up

All patients were followed-up at the University outpatient clinic every 6 months, or earlier if it was clinically required. The primary endpoint was hospitalization due to HF exacerbation (worsening signs of LV or right ventricular failure resulting in administration of intravenous diuretics) including those with fatal outcome (EVENT). The first episode of the EVENT was the end of follow-up period for a given patient. None of the patients were lost during the follow up.

### Statistics

Continuous data are presented as the median (25^th^ - 75^th^ percentiles), or numbers (n) and percentages (%). Comparisons between EVENT(+) and EVENT(-) group were made with the Fisher's exact test, U Mann-Whitney’s test or paired Student's t-test. An association between the analyzed parameters and the endpoint was assessed using the Cox hazard models. The accuracy of pre-specified cut-off values for analyzed parameters and their association as potential predictors of the study endpoint was determined by area (AUC) under the receiver-operating characteristic (ROC) curve. The time course of the endpoint, stratified according to the established cut-off value for GLS, was estimated using the Kaplan-Meier method, and the association between compared groups was estimated by the log-rank test. All the results were considered statistically significant at p ≤ 0.05. The statistical analysis was conducted with STATISTICA 9.0 (*StatSoft*, *Tulsa OK*, *USA*) and R 2.15.2.

## Results

### Clinical characteristics of the studied patients

Finally, 193 patients who met the inclusion criteria were enrolled. The average age of patients was 64 (58–72) years, most of them were men (92%) and 91% underwent myocardial infarction, average LVEF was 33% and GLS -12.3%. Concomitant diseases such as diabetes, hypertension, chronic kidney disease (in stage III or higher) and hypercholesterolemia were present in 53 (27%), 131 (68%), 43 (23%) and 132 (68%), respectively. Regarding pharmacotherapy, a significant proportion of patients received an optimal medical therapy. Baseline clinical, demographic and echocardiographic characteristics of the entire study group are presented in Table 1.

**Table 1:** Clinical, laboratory and echocardiographic characteristics of the study group and comparison between the EVENT(+) and EVENT(-) groups.

	All n = 193	EVENT(+) n = 58	EVENT(-) n = 135	*p
Age (years)	64 (58–72)	67 (59–75)	64 (58–70)	< 0.044
Male, n (%)	177 (92)	53 (91)	124 (92)	1.000
Myocardial infarction, n (%)	175 (91)	51 (88)	124 (92)	0.423
Revascularization, n (%)	173 (90)	53 (91)	120 (89)	0.798
QRS (ms)	90 (110–130)	120 (100–160)	110 (90–120)	< 0.001
ICD, n (%)	115 (60)	50 (86)	65 (48)	< 0.001
CRT-D, n (%)	17 (9)	10 (17)	7 (5)	< 0.011
*NYHA class*				< 0.001
NYHA I, n (%)	34 (18)	5 (9)	29 (21)	
NYHA II, n (%)	127 (66)	35 (60)	92 (68)	
NYHA III, n (%)	32 (17)	18 (30)	14 (10)	
*Laboratory parameters*				
Hemoglobin (g/dl)	14 (13–15)	13.8 (12.6–14.5)	14.1 (13.7–14.8)	<0.049
BNP (pg/ml)	108 (77–300)	232 (104–1046)	104 (64–200)	< 0.018
*Echocardiographic parameters*				
LADs (mm)	45 (41–48)	46 (43–50)	44 (40–48)	< 0.005
EDV (ml)	157 (125–200)	170 (136–243)	153 (114–197)	< 0.029
ESV (ml)	99 (77–134)	120 (96–171)	88 (69–127)	< 0.001
LVEF (%)	33 (27–40)	28 (23–33)	35 (30–40)	< 0.001
GLS (%)	-12.3 (-14.9 –-9.7)	-8.4 (-8.9 –-7.6)	-12.6 (-14.9 –-10.3)	< 0.009
*Medications*				
beta-adrenolytics, n (%)	184 (95)	52 (90)	132 (98)	< 0.023
ACE-inhibitor or ARB, n (%)	178 (92)	55 (95)	123 (91)	0.559
spironolactone/eplerenone, n (%)	100 (52)	31 (53)	69 (51)	0.875
antiplatelet therapy, n (%)	175 (91)	51 (88)	124 (92)	0.423
statins, n (%)	174 (90)	55 (95)	119 (88)	0.193
digoxin, n (%)	6 (3)	5 (9)	1 (1)	< 0.010
diuretics, n (%)	89 (46)	38 (66)	51 (38)	< 0.001
*Concomitant diseases*				
Arterial hypertension, n (%)	131 (68)	41 (71)	90 (67)	0.618
Diabetes, n (%)	53 (27)	26 (45)	27 (20)	< 0.001
GFR < 60 ml/min/1.73 m^2^, n (%)	43 (22)	25 (43)	18 (13)	< 0.001
Hypercholesterolemia, n (%)	132 (68)	39 (67)	93 (69)	0.867
History of atrial fibrillation/flutter, n (%)	41 (21)	17 (29)	24 (18)	0.085
Smoking, n (%)	142 (74)	44 (76)	98 (73)	0.723

Abbreviations: ACE–angiotensin converting enzyme, ARB–angiotensin receptor blocker; BNP–brain natriuretic peptide; CRT-D–implantable cardioverter defibrillator with cardiac resynchronization therapy; LADs–left atrium diameter; LVEF–left ventricular ejection fraction; EDV–left ventricular end-diastolic volume; ESV–left ventricular end-systolic volume; GFR–glomerular filtration rate; GLS–global longitudinal strain; ICD–implantable cardioverter defibrillator; MI–myocardial infarction; NYHA–classification according the New York Heart Association; QRS–QRS complex width

*p value for comparison between EVENT(+) and EVENT(-) groups

**Fig 1:**
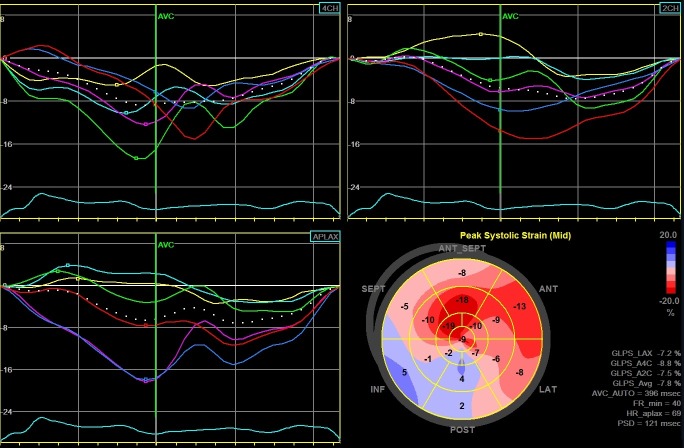
The example of global LV longitudinal strain measurements from long-axis, 2- and 4-chumber views, and „Bull’s-eye” representation of regional strains in patient who was hospitalized due to HF decompensation. Average GLS is -7.8%.

**Fig 2:**
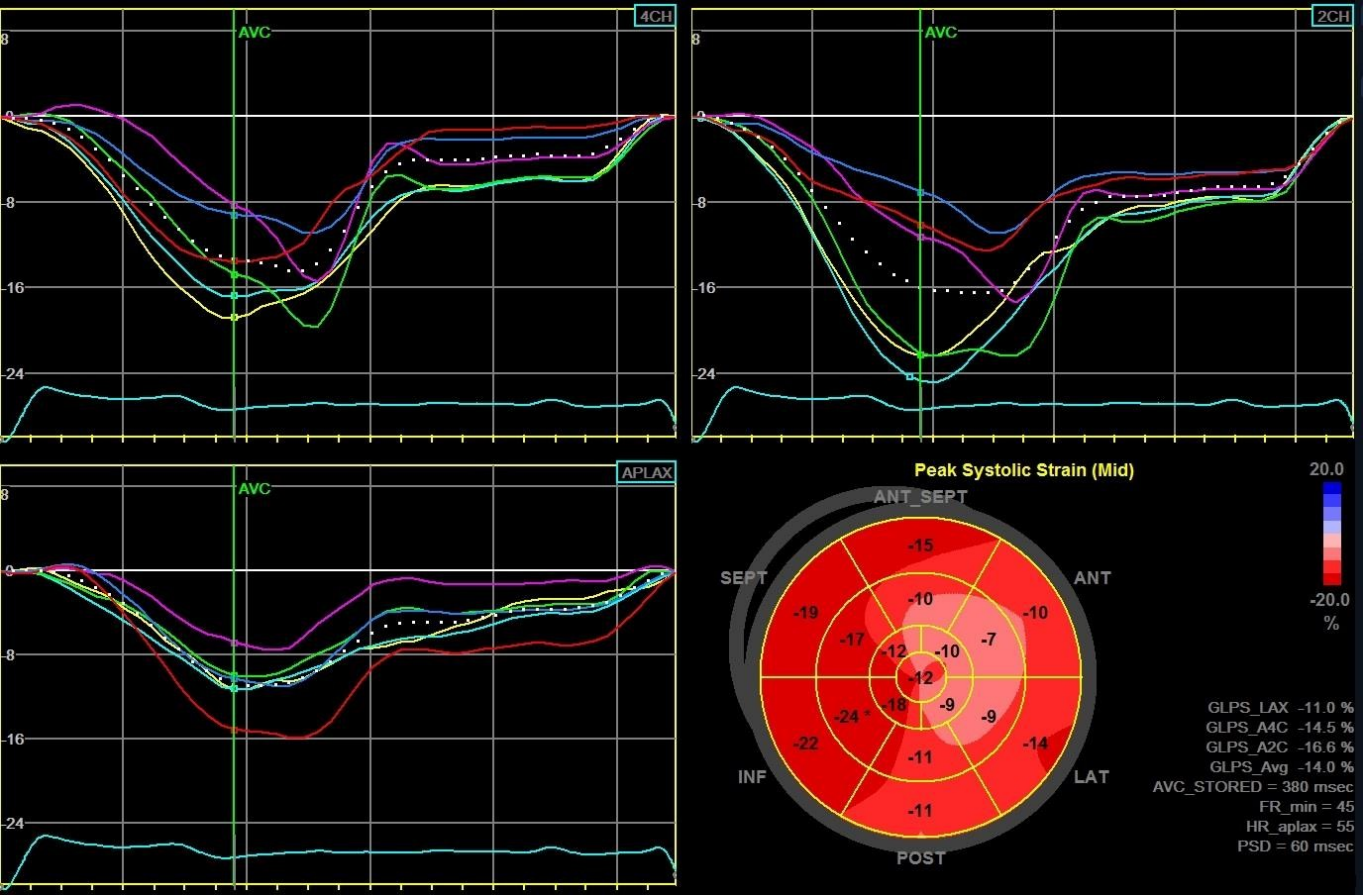
The example of global LV longitudinal strain measurements from long-axis, 2- and 4-chumber views, and „Bull’s-eye” representation of regional strains in patient who didn’t reach the study endpoint within follow-up period.

During 34 (14–71) month follow-up, 58 patients (30%) reached the study endpoint. Patients from the EVENT(+) group were older, had a wider QRS complex, more advanced NYHA functional class, lower hemoglobin, higher brain natriuretic peptide (BNP) level, they were more likely to have diabetes, chronic kidney disease, they less likely used beta-blockers but more often diuretics and digoxin. With regard to the echocardiographic examination, EVENT(+) patients had larger LADs, greater EDV and ESV as well as lower LVEF and worse GLS: respectively -8.4% (-8.9 –-7.6) versus -12.6% (-14.9 –-10.3) in EVENT(-) group (p < 0.009). Fig 1 illustrates the example of GLS measurements with severely decreased average GLS value of -7.8% in patient who was hospitalized due to HF decompensation. Fig 2 presents the example of GLS measurements with moderately reduced average GLS value of -14% in patient who didn't reach study endpoint within follow-up period.

### Predictors of hospitalization due to HF decompensation

Univariate Cox analyses revealed age, QRS, NYHA functional class III, hemoglobin, BNP, LADs, EDV, ESV, LVEF, GLS, absence of beta-blockers, use of diuretics, diabetes and GFR < 60 ml/min/1.73 m^2^ as significant predictors of the EVENT. Pre-specified cut-off values with the highest risk of the EVENT were: age ≥ 72 years, QRS ≥ 118 ms, hemoglobin < 14.8 g/dl, BNP ≥ 214 pg/ml, LAD ≥ 45 mm, EDV ≥ 166 ml, ESV ≥ 89 ml, LVEF < 32%, GLS ≥ -9.4% (Table 2).

**Table 2:** Results of Cox proportional hazard regression analysis for analyzed parameters and their pre-specified cut-off values as predictors of the EVENT during follow-up period.

Parameter	Univariate analysis		*p
	HR	95% CI	
Age (years)	1.05	1.01–1.08	< 0.005
Age ≥ 72 (years)	2.48	1.46–4.23	< 0.001
QRS (ms)	1.01	1–1.02	< 0.004
QRS ≥ 118 (ms)	1.99	1.14–3.49	< 0.016
NYHA class III	2.35	1.34–4.13	< 0.003
Hemoglobin (g/dl)	0.82	0.69–0.98	< 0.029
Hemoglobin < 14.8 (g/dl)	3.02	1.19–7.7	< 0.002
BNP (pg/ml)	1.00	1–1	< 0.004
BNP ≥ 214 (pg/ml)	2.64	1.13–5.31	< 0.006
LAD (mm)	1.07	1.02–1.12	< 0.004
LAD ≥ 45 (mm)	2.08	1.18–3.68	< 0.011
EDV (ml)	1.01	1–1.01	< 0.021
EDV ≥ 166 (ml)	1.83	0.91–3.69	< 0.089
ESV (ml)	1.01	1–1.01	< 0.013
ESV ≥ 89 (ml)	2.64	1.01–6.87	< 0.047
LVEF (%)	0.93	0.9–0.96	< 0.001
LVEF < 32 (%)	2.76	1.55–4.9	< 0.001
GLS (%)	1.32	1–1.74	< 0.049
GLS ≥ -9.4 (%)	15.16	1.81–126.91	< 0.012
Abscence of betablockers	4.21	1.76–10.9	< 0.001
Diuretics	2.17	1.26–3.73	< 0.005
Diabetes	2.45	1.46–4.14	< 0.001
GFR < 60 ml/min/1.73 m^2^	3.16	1.87–5.34	< 0.001

Abbreviations: BNP–brain natriuretic peptide; CI–confidence interval; LADs–left atrium diameter; LVEF–left ventricular ejection fraction; EDV–left ventricular end-diastolic volume; ESV–left ventricular end-systolic volume; GFR–glomerular filtration rate; GLS–global longitudinal strain; HR–hazard ratio; NYHA–classification according the New York Heart Association; QRS–QRS complex width

*p value for comparison between EVENT(+) and EVENT(-) groups

ROC analysis identified GLS as the most accurate predictor of hospitalization due to HF decompensation among all the studied continuous variables (AUC 0.81, hazard ratio [HR] 15.16, 95% confidence interval [CI] 1.81–126.91, p < 0.012) with high positive predictive value (PPV) of 98%, whereas the rest of the analyzed parameters were characterized by lower discriminatory powers as presented in Table 3.

**Table 3:** Prognostic accuracy of the pre-specified cut-off values for analyzed parameters as the predictors of the EVENT during the follow-up.

Parameters	AUC	Characteristics (95% CI)		Predictive Value (95% CI)	
		Sensitivity (%)	Specificity (%)	Positive (%)	Negative (%)
Age ≥ 72 (years)	0.59	80	40	76	46
QRS ≥ 118 (ms)	0.67	61	68	82	43
Hemoglobin < 14.8 (g/dl)	0.59	32	88	85	37
BNP ≥ 214 (pg/ml)	0.73	79	56	77	58
LADs ≥ 45 (mm)	0.63	54	67	78	41
EDV ≥ 166 (ml)	0.63	67	58	77	44
ESV ≥ 89 (ml)	0.72	51	85	88	44
LVEF < 32 (%)	0.76	67	72	85	49
**GLS ≥ -9.4 (%)**	**0.81**	**87**	**86**	**98**	**50**

Abbreviations: AUC–area under curve; BNP–brain natriuretic peptide; CI–confidence interval; HGB–hemoglobin; LADs–left atrium diameter; LVEF–left ventricular ejection fraction; EDV–left ventricular end-diastolic volume; ESV–left ventricular end-systolic volume; GFR–glomerular filtration rate; GLS–global longitudinal strain; QRS–QRS complex width

**Fig 3:**
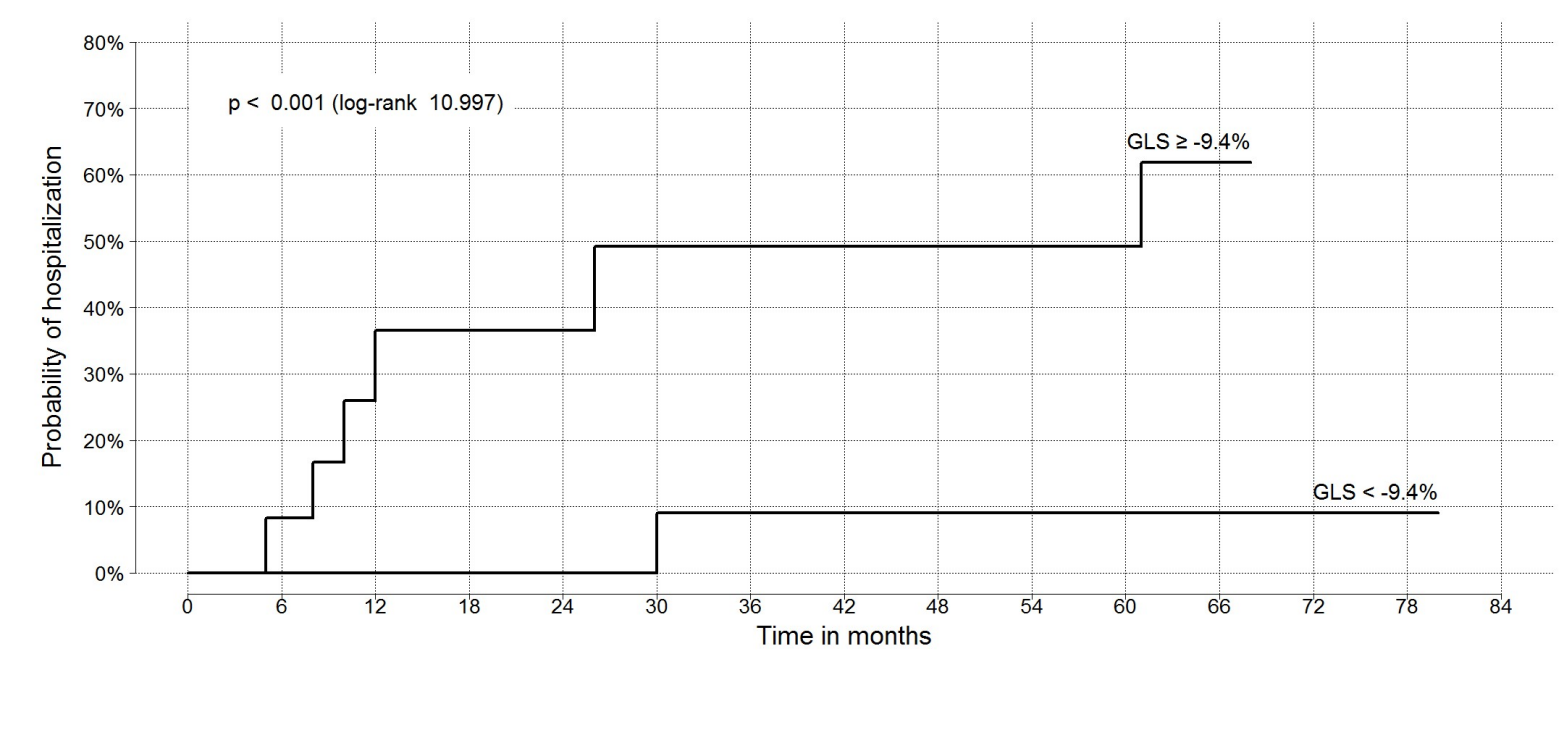
Kaplan-Meier curves illustrating probability of the hospitalization due to HF exacerbation after dichotomization according the pre-specified cut-off values for GLS during follow-up period.

GLS maintained its statistical significance as a predictor of the EVENT even after adjusting to other above-mentioned parameters. Fig 3 illustrates Kaplan-Meier’s curve with the probability of the hospitalization due to HF exacerbation depending on pre-specified cut-off values for GLS during follow-up period.

## Discussion

In this study, we proved that GLS with a cut-off value of ≥ -9.4% is a statistically significant indicator of hospitalization due to HF decompensation, independent of other known risk factors in a well-defined group of stable individuals with ischemic cardiomyopathy with LVEF up to 50% who were optimally treated. In our opinion, assessment of GLS in clinical practice seems to be useful in identifying which patients should receive expanded ambulatory care to reduce the risk of HF exacerbations.

The role of GLS as a marker of cardiovascular events has been studied extensively in different groups of patients with regard to various endpoints [16, 27–30]. For example, in the study by Cho G et al., GLS was proved to be a predictor of the composite endpoint (cardiac mortality and HF exacerbations) in acute HF patients [27]. Nahum J et al., established the usefulness of GLS in the patients with chronic HF, but contrary to our study, they included patients with diverse HF etiology [28]. Buggey J et al., demonstrated the relationship between GLS and mortality or hospitalization for HF exacerbation in patients with acute HF and preserved EF (≥ 50%), however, this relationship was noted only in short-term observation [29].

GLS cut-off value of ≥ -9.4% determined in the present study in HF patients with LVEF up to 50% is within the range of values ​​presented by other authors, varying from about -7% to -14% [13, 31–34]. The value of this parameter is usually better in groups of patients with higher LVEF and worse in those with lower LVEF.

Particularly noteworthy is that GLS cut-off value established in the present study was characterized by the highest discriminatory power in revealing the risk of HF decompensation. AUC for GLS ≥ -9.4% was 0.81, while for broadly acknowledge LVEF it was lower (0.76). Furthermore, in the case of GLS values ​​≥ -9.4%, the relative risk of hospitalization due to HF decompensation was 15 times higher, while for others at most 2–3 fold. It is also worth to emphasize the very high PPV of GLS (98%), which was the highest PPV of all analyzed parameters. Sensitivity and specificity of this parameter were also high (87% and 86%, respectively). Thereby, GLS proved to be the strongest indicator of HF decompensation in the group of stable patients with ischemic cardiomyopathy with LVEF ≤ 50%. In addition, it is a quick and easy to obtain parameter, even at the patient's bedside, more objective and not based on geometrical assumptions, as in case of LVEF. GLS seems to be considered as a standard parameter evaluated in patients with HF for identification the most at-risk persons requiring special care.

## Study limitations

It was a small, single-center investigation and due to the relatively small number of patients, it was impossible to create a more stable subpopulation of patients who reached the endpoint, and thus the results need to be confirmed in a larger group of patients. Next, functional performance of the studied population was not evaluated [35] (for example by 6 minute walking test) and patients permanent atrial fibrillation were excluded from the study. Furthermore, other speckle tracking parameters, such as circumferential and radial strain, which are shown to be promising, were not evaluated [36]. Moreover, GLS was assessed using only one of the available vendor platforms, therefore the results may be slightly different from those received with alternative software algorithms [37].

## Conclusions

GLS derived by 2D STE can provide, independently of other well-known clinical parameters, information on the risk of hospitalization due to HF decompensation in the stable outpatients with LV systolic dysfunction of ischemic origin. Decreased GLS with cut-off value of ≥ -9.4% can identify the patients with the highest risk of such event, who require special care.

## Supporting information

S1 File Data set. (PDF)
