# Supplementary material for: Global longitudinal strain can predict heart failure exacerbation in stable outpatients with ischemic left ventricular systolic dysfunction
Source: PLoS One. 2019 Dec 2;14(12):e0225829. doi: 10.1371/journal.pone.0225829 (PMC6886774; doi:10.1371/journal.pone.0225829)
Supplement: S1 File — (PDF) [file pone.0225829.s001.pdf]

Ordinal nu	Age (years	Gender	Hospitalization due HF exacerbation	LVEF	NYHA	NYHA divis
1	63	M	0	40	2	NYHA_12
2	66	M	1	38	2	NYHA_12
3	69	M	1	30	1	NYHA_12
4	64	M	1	30	2	NYHA_12
5	59	K	1	21	3	NYHA_3
6	65	M	0	25	2	NYHA_12
7	68	K	0	33	2	NYHA_12
8	61	M	0	35	1	NYHA_12
9	52	K	0	35	2	NYHA_12
10	44	K	0	25	2	NYHA_12
11	66	M	0	42	1	NYHA_12
12	69	M	0	38	1	NYHA_12
13	85	M	1	28	2	NYHA_12
14	68	M	0	32	2	NYHA_12
15	89	M	0	35	3	NYHA_3
16	61	M	1	38	2	NYHA_12
17	60	M	0	50	1	NYHA_12
18	71	M	0	33	1	NYHA_12
19	56	M	0	35	2	NYHA_12
20	59	M	0	35	2	NYHA_12
21	73	M	0	47	1	NYHA_12
22	75	M	0	35	2	NYHA_12
23	59	M	0	37	2	NYHA_12
24	70	M	1	37	2	NYHA_12
25	51	M	1	39	1	NYHA_12
26	74	M	0	30	2	NYHA_12
27	47	K	0	35	2	NYHA_12
28	54	M	1	28	2	NYHA_12
29	70	M	0	40	2	NYHA_12
30	64	M	0	30	2	NYHA_12
31	50	M	0	40	1	NYHA_12
32	67	M	1	26	2	NYHA_12
33	52	M	0	26	2	NYHA_12
34	79	M	0	39	2	NYHA_12
35	61	M	1	30	2	NYHA_12
36	76	M	0	26	2	NYHA_12
37	66	M	0	45	2	NYHA_12
38	70	M	0	45	1	NYHA_12
39	73	K	0	33	3	NYHA_3
40	60	M	0	48	2	NYHA_12
41	64	M	0	47	2	NYHA_12
42	54	M	0	40	2	NYHA_12
43	60	M	0	31	2	NYHA_12
44	65	M	0	43	2	NYHA_12
45	58	M	1	23	3	NYHA_3
46	77	M	1	20	2	NYHA_12
47	65	M	1	35	2	NYHA_12
48	57	K	0	37	3	NYHA_3
49	75	M	1	32	3	NYHA_3

50	69	M	0	39	2	NYHA_12
51	71	M	0	38	1	NYHA_12
52	75	M	1	30	3	NYHA_3
53	76	M	1	30	3	NYHA_3
54	61	M	0	45	3	NYHA_3
55	54	M	0	35	1	NYHA_12
56	57	M	0	32	1	NYHA_12
57	72	M	1	28	3	NYHA_3
58	53	M	0	27	2	NYHA_12
59	60	M	0	38	2	NYHA_12
60	59	M	0	35	1	NYHA_12
61	79	K	0	48	2	NYHA_12
62	65	M	0	32	2	NYHA_12
63	70	M	0	47	2	NYHA_12
64	74	M	1	21	3	NYHA_3
65	76	K	1	25	3	NYHA_3
66	75	M	0	26	3	NYHA_3
67	74	M	0	38	1	NYHA_12
68	56	M	0	40	2	NYHA_12
69	61	K	0	28	3	NYHA_3
70	60	M	0	50	2	NYHA_12
71	58	M	0	45	2	NYHA_12
72	61	M	0	48	1	NYHA_12
73	38	M	0	20	3	NYHA_3
74	50	M	0	35	2	NYHA_12
75	64	M	0	25	2	NYHA_12
76	47	M	0	36	2	NYHA_12
77	63	M	1	35	2	NYHA_12
78	54	M	1	19	3	NYHA_3
79	73	M	0	30	2	NYHA_12
80	81	M	0	26	2	NYHA_12
81	78	M	0	30	2	NYHA_12
82	58	M	1	20	2	NYHA_12
83	56	M	1	42	2	NYHA_12
84	54	M	1	23	2	NYHA_12
85	20	K	0	45	2	NYHA_12
86	75	M	1	40	2	NYHA_12
87	71	M	1	22	2	NYHA_12
88	63	M	0	36	2	NYHA_12
89	63	M	0	19	2	NYHA_12
90	59	M	0	28	2	NYHA_12
91	54	M	1	10	3	NYHA_3
92	69	M	1	25	2	NYHA_12
93	65	M	0	35	2	NYHA_12
94	78	M	1	37	1	NYHA_12
95	60	M	1	27	1	NYHA_12
96	57	M	0	30	1	NYHA_12
97	68	M	0	35	2	NYHA_12
98	63	M	0	43	1	NYHA_12
99	56	K	1	30	2	NYHA_12

100	73	M	0	45	2	NYHA_12
101	60	M	0	30	1	NYHA_12
102	71	M	0	30	2	NYHA_12
103	76	M	1	35	2	NYHA_12
104	71	M	0	36	2	NYHA_12
105	70	M	0	33	2	NYHA_12
106	58	M	1	32	2	NYHA_12
107	67	M	0	39	2	NYHA_12
108	58	M	0	28	2	NYHA_12
109	60	M	1	33	2	NYHA_12
110	75	M	0	42	2	NYHA_12
111	65	M	0	42	2	NYHA_12
112	76	M	1	39	2	NYHA_12
113	75	M	0	40	2	NYHA_12
114	64	M	1	20	2	NYHA_12
115	50	M	0	30	1	NYHA_12
116	76	M	1	33	2	NYHA_12
117	65	M	0	42	2	NYHA_12
118	61	M	0	20	2	NYHA_12
119	74	M	1	32	3	NYHA_3
120	64	M	1	23	2	NYHA_12
121	62	M	0	48	1	NYHA_12
122	77	M	0	35	3	NYHA_3
123	57	M	0	49	2	NYHA_12
124	70	M	0	35	2	NYHA_12
125	79	M	1	33	2	NYHA_12
126	58	M	1	17	3	NYHA_3
127	65	K	0	40	2	NYHA_12
128	51	M	0	43	2	NYHA_12
129	60	M	0	33	1	NYHA_12
130	63	M	1	31	2	NYHA_12
131	58	M	1	28	2	NYHA_12
132	70	M	0	23	2	NYHA_12
133	57	M	1	28	2	NYHA_12
134	69	M	0	32	2	NYHA_12
135	67	M	0	42	2	NYHA_12
136	51	M	0	46	1	NYHA_12
137	59	M	0	35	3	NYHA_3
138	76	M	0	30	1	NYHA_12
139	72	M	0	37	1	NYHA_12
140	60	M	0	46	3	NYHA_3
141	47	M	1	19	3	NYHA_3
142	52	M	0	27	3	NYHA_3
143	62	M	0	33	2	NYHA_12
144	57	M	0	47	2	NYHA_12
145	82	M	1	25	3	NYHA_3
146	60	M	0	40	1	NYHA_12
147	80	M	0	38	3	NYHA_3
148	85	M	0	40	2	NYHA_12
149	64	M	0	43	2	NYHA_12

150	69	M	0	35	2	NYHA_12
151	61	M	0	20	2	NYHA_12
152	57	M	0	30	3	NYHA_3
153	68	M	0	18	1	NYHA_12
154	81	M	0	40	2	NYHA_12
155	54	M	0	38	2	NYHA_12
156	57	M	0	40	2	NYHA_12
157	65	M	0	25	2	NYHA_12
158	73	M	1	25	2	NYHA_12
159	67	K	0	17	3	NYHA_3
160	74	M	0	25	2	NYHA_12
161	56	M	0	20	2	NYHA_12
162	74	M	0	40	2	NYHA_12
163	50	M	0	27	2	NYHA_12
164	72	K	1	35	3	NYHA_3
165	59	K	1	13	2	NYHA_12
166	46	M	0	35	1	NYHA_12
167	71	M	0	46	1	NYHA_12
168	74	M	0	28	2	NYHA_12
169	75	M	1	27	3	NYHA_3
170	64	M	1	15	2	NYHA_12
171	54	M	0	24	2	NYHA_12
172	66	M	0	35	2	NYHA_12
173	65	M	0	40	1	NYHA_12
174	75	M	0	40	2	NYHA_12
175	63	M	1	20	3	NYHA_3
176	63	M	0	45	2	NYHA_12
177	59	M	0	35	2	NYHA_12
178	70	M	1	30	2	NYHA_12
179	77	M	1	25	2	NYHA_12
180	72	M	0	27	2	NYHA_12
181	64	M	0	32	2	NYHA_12
182	70	M	0	38	2	NYHA_12
183	61	M	0	32	2	NYHA_12
184	58	M	0	38	2	NYHA_12
185	58	M	0	27	1	NYHA_12
186	58	M	0	49	2	NYHA_12
187	65	M	0	33	2	NYHA_12
188	73	M	1	17	2	NYHA_12
189	51	M	0	45	2	NYHA_12
190	57	M	0	42	2	NYHA_12
191	74	M	1	45	1	NYHA_12
192	66	M	0	33	2	NYHA_12
193	77	M	1	23	3	NYHA_3

CAD	MI	Revascular	History of a	Arterial Hy	Diabetes	GFR.Divisio	GFR.Divisio	GFR.Divisio
CAD+	MI+	1	0	1	0	GFR<60	GFR 30-59	GFR>30
CAD+	MI+	1	0	1	0	GFR<60	GFR 30-59	GFR>30
CAD+	MI+	1	0	1	0	GFR>60	GFR>60	GFR>30
CAD+	MI+	1	0	1	0	GFR<60	GFR 30-59	GFR>30
CAD+	MI+	1	0	1	0	GFR>60	GFR>60	GFR>30
CAD+	MI+	1	0	1	0	GFR>60	GFR>60	GFR>30
CAD+	MI+	1	0	1	1	GFR>60	GFR>60	GFR>30
CAD+	MI+	1	0	1	1	GFR>60	GFR>60	GFR>30
CAD+	MI+	1	0	1	0	GFR>60	GFR>60	GFR>30
CAD+	MI+	1	0	1	1	GFR>60	GFR>60	GFR>30
CAD+	MI-	1	0	0	0	GFR>60	GFR>60	GFR>30
CAD+	MI-	1	0	1	0	GFR>60	GFR>60	GFR>30
CAD+	MI+	1	0	1	0	GFR<60	GFR 30-59	GFR>30
CAD+	MI+	1	0	1	0	GFR>60	GFR>60	GFR>30
CAD+	MI+	1	0	1	0	GFR>60	GFR>60	GFR>30
CAD+	MI-	0	0	0	0	GFR>60	GFR>60	GFR>30
CAD+	MI+	1	0	1	0	GFR>60	GFR>60	GFR>30
CAD+	MI+	1	0	0	0	GFR>60	GFR>60	GFR>30
CAD+	MI+	1	0	1	0	GFR>60	GFR>60	GFR>30
CAD+	MI+	1	0	1	0	GFR>60	GFR>60	GFR>30
CAD+	MI+	1	0	0	0	GFR>60	GFR>60	GFR>30
CAD+	MI+	1	1	1	0	GFR<60	GFR 30-59	GFR>30
CAD+	MI+	1	0	1	1	GFR>60	GFR>60	GFR>30
CAD+	MI+	1	0	1	0	GFR>60	GFR>60	GFR>30
CAD+	MI+	1	1	0	0	GFR>60	GFR>60	GFR>30
CAD+	MI+	1	0	0	0	GFR>60	GFR>60	GFR>30
CAD+	MI+	1	0	1	0	GFR>60	GFR>60	GFR>30
CAD+	MI+	1	0	0	0	GFR>60	GFR>60	GFR>30
CAD+	MI+	1	1	1	0	GFR>60	GFR>60	GFR>30
CAD+	MI+	1	0	0	1	GFR>60	GFR>60	GFR>30
CAD+	MI+	1	0	0	0	GFR>60	GFR>60	GFR>30
CAD+	MI-	1	0	1	0	GFR>60	GFR>60	GFR>30
CAD+	MI+	1	0	1	0	GFR>60	GFR>60	GFR>30
CAD+	MI+	1	1	1	0	GFR>60	GFR>60	GFR>30
CAD+	MI+	1	1	1	1	GFR<60	GFR 30-59	GFR>30
CAD+	MI+	1	0	1	0	GFR<60	GFR 30-59	GFR>30
CAD+	MI+	1	0	1	0	GFR<60	GFR 30-59	GFR>30
CAD+	MI+	1	0	1	0	GFR<60	GFR 30-59	GFR>30
CAD+	MI+	1	0	1	0	GFR<60	GFR 30-59	GFR>30
CAD+	MI+	1	1	1	0	GFR>60	GFR>60	GFR>30
CAD+	MI+	1	1	1	0	GFR>60	GFR>60	GFR>30
CAD+	MI+	1	0	0	0	GFR>60	GFR>60	GFR>30
CAD+	MI+	1	0	1	0	GFR>60	GFR>60	GFR>30
CAD+	MI+	1	0	1	1	GFR>60	GFR>60	GFR>30
CAD+	MI+	1	1	1	0	GFR<60	GFR 30-59	GFR>30
CAD+	MI+	1	0	1	1	GFR>60	GFR>60	GFR>30
CAD+	MI+	1	1	1	1	GFR<60	GFR 30-59	GFR>30
CAD+	MI+	1	0	1	0	GFR>60	GFR>60	GFR>30
CAD+	MI+	1	1	1	1	GFR>60	GFR>60	GFR>30

CAD+	MI+	1	0	1	0	GFR>60	GFR>60	GFR>30
CAD+	MI+	1	0	0	0	GFR>60	GFR>60	GFR>30
CAD+	MI+	1	0	1	1	GFR>60	GFR>60	GFR>30
CAD+	MI+	0	0	1	0	GFR>60	GFR>60	GFR>30
CAD+	MI+	1	0	1	0	GFR>60	GFR>60	GFR>30
CAD+	MI+	1	1	0	0	GFR>60	GFR>60	GFR>30
CAD+	MI+	1	0	1	1	GFR>60	GFR>60	GFR>30
CAD+	MI+	0	0	1	0	GFR>60	GFR>60	GFR>30
CAD+	MI+	1	0	0	0	GFR>60	GFR>60	GFR>30
CAD+	MI+	1	0	1	0	GFR>60	GFR>60	GFR>30
CAD+	MI+	1	0	0	0	GFR>60	GFR>60	GFR>30
CAD+	MI+	1	0	1	1	GFR<60	GFR 30-59	GFR>30
CAD+	MI+	0	1	0	1	GFR>60	GFR>60	GFR>30
CAD+	MI+	1	1	1	1	GFR>60	GFR>60	GFR>30
CAD+	MI+	1	0	1	1	GFR<60	GFR 30-59	GFR>30
CAD+	MI+	1	1	1	1	GFR<60	GFR<30	GFR<30
CAD+	MI+	1	0	0	0	GFR>60	GFR>60	GFR>30
CAD+	MI+	1	0	1	0	GFR>60	GFR>60	GFR>30
CAD+	MI+	1	0	0	0	GFR>60	GFR>60	GFR>30
CAD+	MI+	1	0	1	1	GFR>60	GFR>60	GFR>30
CAD+	MI+	1	0	1	1	GFR>60	GFR>60	GFR>30
CAD+	MI+	1	0	0	0	GFR>60	GFR>60	GFR>30
CAD+	MI+	1	0	1	0	GFR>60	GFR>60	GFR>30
CAD+	MI+	1	0	0	0	GFR>60	GFR>60	GFR>30
CAD+	MI+	1	0	1	0	GFR>60	GFR>60	GFR>30
CAD+	MI-	1	1	1	0	GFR<60	GFR 30-59	GFR>30
CAD+	MI+	1	0	0	0	GFR>60	GFR>60	GFR>30
CAD+	MI+	1	0	1	1	GFR>60	GFR>60	GFR>30
CAD+	MI+	1	0	1	0	GFR<60	GFR 30-59	GFR>30
CAD+	MI+	1	1	0	0	GFR<60	GFR 30-59	GFR>30
CAD+	MI+	1	0	0	0	GFR>60	GFR>60	GFR>30
CAD+	MI+	1	0	1	0	GFR>60	GFR>60	GFR>30
CAD+	MI+	1	0	0	0	GFR<60	GFR<30	GFR<30
CAD+	MI+	1	0	1	1	GFR<60	GFR 30-59	GFR>30
CAD+	MI+	1	1	1	1	GFR<60	GFR 30-59	GFR>30
CAD+	MI+	0	0	0	0	GFR>60	GFR>60	GFR>30
CAD+	MI+	1	0	1	1	GFR<60	GFR 30-59	GFR>30
CAD+	MI+	1	0	1	1	GFR>60	GFR>60	GFR>30
CAD+	MI+	0	0	1	0	GFR>60	GFR>60	GFR>30
CAD+	MI+	1	0	0	0	GFR<60	GFR 30-59	GFR>30
CAD+	MI+	1	1	1	0	GFR>60	GFR>60	GFR>30
CAD+	MI-	1	0	0	0	GFR>60	GFR>60	GFR>30
CAD+	MI+	1	0	1	0	GFR>60	GFR>60	GFR>30
CAD+	MI+	1	1	1	1	GFR>60	GFR>60	GFR>30
CAD+	MI+	1	1	1	0	GFR>60	GFR>60	GFR>30
CAD+	MI+	1	0	1	0	GFR>60	GFR>60	GFR>30
CAD+	MI+	1	0	1	0	GFR>60	GFR>60	GFR>30
CAD+	MI+	1	0	1	1	GFR>60	GFR>60	GFR>30
CAD+	MI+	1	0	1	0	GFR>60	GFR>60	GFR>30
CAD+	MI-	0	1	0	0	GFR>60	GFR>60	GFR>30

CAD+	MI-	0	1	1	0	GFR>60	GFR>60	GFR>30
CAD+	MI+	1	0	0	0	GFR>60	GFR>60	GFR>30
CAD+	MI+	1	0	0	0	GFR>60	GFR>60	GFR>30
CAD+	MI+	0	0	0	0	GFR<60	GFR 30-59	GFR>30
CAD+	MI+	1	0	0	1	GFR>60	GFR>60	GFR>30
CAD+	MI+	0	0	0	0	GFR>60	GFR>60	GFR>30
CAD+	MI+	1	0	1	1	GFR>60	GFR>60	GFR>30
CAD+	MI+	1	0	1	0	GFR>60	GFR>60	GFR>30
CAD+	MI+	1	0	1	1	GFR>60	GFR>60	GFR>30
CAD+	MI+	1	1	1	0	GFR<60	GFR 30-59	GFR>30
CAD+	MI+	1	0	1	0	GFR>60	GFR>60	GFR>30
CAD+	MI+	0	0	1	0	GFR>60	GFR>60	GFR>30
CAD+	MI+	1	0	1	1	GFR>60	GFR>60	GFR>30
CAD+	MI-	1	0	0	0	GFR<60	GFR 30-59	GFR>30
CAD+	MI+	1	0	1	0	GFR>60	GFR>60	GFR>30
CAD+	MI+	1	0	1	0	GFR>60	GFR>60	GFR>30
CAD+	MI+	1	0	1	0	GFR>60	GFR>60	GFR>30
CAD+	MI+	1	0	0	0	GFR>60	GFR>60	GFR>30
CAD+	MI+	1	0	1	0	GFR>60	GFR>60	GFR>30
CAD+	MI+	1	1	0	1	GFR<60	GFR 30-59	GFR>30
CAD+	MI+	1	0	1	1	GFR>60	GFR>60	GFR>30
CAD+	MI-	0	0	1	0	GFR>60	GFR>60	GFR>30
CAD+	MI-	1	1	1	0	GFR<60	GFR 30-59	GFR>30
CAD+	MI+	1	1	1	0	GFR>60	GFR>60	GFR>30
CAD+	MI+	0	0	1	1	GFR>60	GFR>60	GFR>30
CAD+	MI+	1	0	1	1	GFR>60	GFR>60	GFR>30
CAD+	MI-	1	0	1	1	GFR>60	GFR>60	GFR>30
CAD+	MI+	1	0	1	1	GFR>60	GFR>60	GFR>30
CAD+	MI+	1	0	0	1	GFR>60	GFR>60	GFR>30
CAD+	MI-	0	0	0	0	GFR>60	GFR>60	GFR>30
CAD+	MI+	1	0	1	1	GFR>60	GFR>60	GFR>30
CAD+	MI-	1	0	0	0	GFR>60	GFR>60	GFR>30
CAD+	MI+	1	1	1	0	GFR>60	GFR>60	GFR>30
CAD+	MI+	1	0	1	1	GFR>60	GFR>60	GFR>30
CAD+	MI+	0	0	0	0	GFR<60	GFR 30-59	GFR>30
CAD+	MI+	0	0	1	0	GFR>60	GFR>60	GFR>30
CAD+	MI+	1	0	0	0	GFR>60	GFR>60	GFR>30
CAD+	MI+	1	0	1	0	GFR>60	GFR>60	GFR>30
CAD+	MI+	1	0	1	0	GFR>60	GFR>60	GFR>30
CAD+	MI+	1	1	1	0	GFR>60	GFR>60	GFR>30
CAD+	MI+	1	0	1	0	GFR>60	GFR>60	GFR>30
CAD+	MI+	1	0	0	0	GFR>60	GFR>60	GFR>30
CAD+	MI-	1	0	1	0	GFR>60	GFR>60	GFR>30
CAD+	MI+	1	0	1	1	GFR>60	GFR>60	GFR>30
CAD+	MI+	1	0	0	0	GFR>60	GFR>60	GFR>30
CAD+	MI+	1	1	1	1	GFR<60	GFR<30	GFR<30
CAD+	MI+	1	0	1	0	GFR>60	GFR>60	GFR>30
CAD+	MI+	1	0	1	1	GFR<60	GFR 30-59	GFR>30
CAD+	MI+	1	1	1	1	GFR<60	GFR 30-59	GFR>30
CAD+	MI+	1	0	1	0	GFR>60	GFR>60	GFR>30

CAD+	MI+	1	0	1	0	GFR>60	GFR>60	GFR>30
CAD+	MI-	1	0	1	1	GFR>60	GFR>60	GFR>30
CAD+	MI+	1	0	1	0	GFR>60	GFR>60	GFR>30
CAD+	MI-	0	0	0	0	GFR>60	GFR>60	GFR>30
CAD+	MI+	1	0	1	0	GFR<60	GFR<30	GFR<30
CAD+	MI+	1	1	0	0	GFR>60	GFR>60	GFR>30
CAD+	MI+	1	1	0	0	GFR>60	GFR>60	GFR>30
CAD+	MI+	1	0	0	1	GFR>60	GFR>60	GFR>30
CAD+	MI+	1	0	0	0	GFR<60	GFR 30-59	GFR>30
CAD+	MI+	1	0	0	0	GFR<60	GFR 30-59	GFR>30
CAD+	MI+	1	0	1	0	GFR>60	GFR>60	GFR>30
CAD+	MI+	0	0	1	0	GFR>60	GFR>60	GFR>30
CAD+	MI+	1	0	1	1	GFR>60	GFR>60	GFR>30
CAD+	MI+	1	0	1	0	GFR>60	GFR>60	GFR>30
CAD+	MI+	1	1	0	1	GFR<60	GFR 30-59	GFR>30
CAD+	MI+	1	0	1	0	GFR<60	GFR 30-59	GFR>30
CAD+	MI+	1	0	1	0	GFR>60	GFR>60	GFR>30
CAD+	MI+	1	0	1	0	GFR>60	GFR>60	GFR>30
CAD+	MI+	1	0	0	1	GFR>60	GFR>60	GFR>30
CAD+	MI+	1	0	0	1	GFR<60	GFR 30-59	GFR>30
CAD+	MI+	1	1	0	0	GFR<60	GFR 30-59	GFR>30
CAD+	MI+	1	0	1	0	GFR>60	GFR>60	GFR>30
CAD+	MI+	0	1	0	0	GFR>60	GFR>60	GFR>30
CAD+	MI+	1	0	1	0	GFR>60	GFR>60	GFR>30
CAD+	MI+	1	0	0	0	GFR>60	GFR>60	GFR>30
CAD+	MI+	1	1	1	1	GFR<60	GFR<30	GFR<30
CAD+	MI+	1	0	1	0	GFR>60	GFR>60	GFR>30
CAD+	MI+	1	0	0	0	GFR>60	GFR>60	GFR>30
CAD+	MI+	1	0	0	0	GFR<60	GFR 30-59	GFR>30
CAD+	MI+	1	1	1	1	GFR>60	GFR>60	GFR>30
CAD+	MI+	1	1	1	0	GFR>60	GFR>60	GFR>30
CAD+	MI+	0	0	1	0	GFR>60	GFR>60	GFR>30
CAD+	MI+	1	1	0	1	GFR>60	GFR>60	GFR>30
CAD+	MI+	1	0	1	0	GFR>60	GFR>60	GFR>30
CAD+	MI+	1	0	0	0	GFR>60	GFR>60	GFR>30
CAD+	MI+	1	1	0	0	GFR<60	GFR 30-59	GFR>30
CAD+	MI+	1	0	1	0	GFR>60	GFR>60	GFR>30
CAD+	MI+	1	0	0	0	GFR>60	GFR>60	GFR>30
CAD+	MI+	1	0	0	0	GFR>60	GFR>60	GFR>30
CAD+	MI+	1	0	1	0	GFR>60	GFR>60	GFR>30
CAD+	MI+	1	0	1	0	GFR>60	GFR>60	GFR>30
CAD+	MI+	1	0	1	0	GFR>60	GFR>60	GFR>30
CAD+	MI+	1	0	1	0	GFR>60	GFR>60	GFR>30
CAD+	MI-	1	1	0	1	GFR<60	GFR 30-59	GFR>30

GFR	GFR>60-0,	GFR MDRD	Hypercholo	Digoxin	Beta-adrer	ACEI-inhibi	Spironolac	Diuretics
52	1	52	1	0	1	1	1	0
43	1	43	1	0	1	1	1	0
>60	0	60	1	0	1	1	1	0
53,4	1	53	1	0	1	1	0	0
NA	0	NA	1	0	1	1	1	1
>60	0	>60	1	0	1	1	1	1
>60	0	>60	1	0	1	1	1	0
>60	0	>60	1	0	1	1	1	1
>60	0	>60	1	0	1	1	1	1
>60	0	>60	1	0	1	1	1	1
>60	0	>60	0	0	1	1	1	1
>60	0	>60	1	0	1	1	1	1
31	2	31	0	0	1	1	1	1
>60	0	>60	1	0	1	1	1	1
>60	0	>60	0	0	1	1	0	0
>60	0	>60	1	0	1	1	1	0
>60	0	>60	1	0	1	0	1	0
>60	0	>60	0	0	1	1	0	1
>60	0	>60	1	0	1	1	0	0
>60	0	>60	1	0	1	1	0	0
>60	0	>60	0	0	1	1	0	0
40,6	1	41	1	0	1	1	0	1
>60	0	>60	1	0	1	1	1	0
NA	0	NA	1	0	1	1	0	0
>60	0	>60	1	0	1	1	1	0
>60	0	>60	1	0	1	1	1	1
NA	0	NA	1	0	1	1	1	1
NA	0	NA	0	0	1	1	1	1
>60	0	>60	1	0	1	1	1	1
NA	0	NA	0	0	1	1	1	0
>60	0	>60	0	0	1	1	1	0
>60	0	>60	1	0	1	1	0	1
>60	0	>60	1	0	1	1	0	0
>60	0	>60	1	0	0	1	0	0
<60	1	<60	1	0	1	1	0	0
31,94	1	32	0	0	1	1	1	1
58	1	58	1	0	1	1	0	0
NA	1	NA	1	0	1	1	1	1
56,47	1	56	1	0	1	1	1	1
>60	0	>60	0	0	1	1	1	1
>60	0	>60	1	0	1	1	1	0
>60	0	>60	0	0	1	1	1	0
>60	0	>60	1	0	1	1	1	0
>60	0	>60	0	0	1	0	0	0
40,36	1	40	1	0	1	1	0	1
>60	0	>60	0	0	1	1	1	1
46,23	1	46	1	0	1	1	1	1
>60	0	>60	0	0	1	1	0	0
>60	0	>60	1	1	1	1	1	1

NA	0	NA	1	0	1	1	1	1
NA	0	NA	1	0	1	0	0	0
>60	0	>60	1	0	1	1	0	1
NA	0	NA	0	0	1	1	1	1
>60	0	>60	1	0	1	1	0	1
NA	0	NA	0	0	1	0	1	1
>60	0	>60	1	0	1	1	0	1
>60	0	>60	0	0	1	1	0	0
>60	0	>60	1	0	1	1	0	0
>60	0	>60	1	0	1	1	0	0
>60	0	>60	1	0	1	0	0	0
38	1	38	1	0	1	1	0	1
>60	0	>60	0	0	1	1	0	0
>60	0	>60	0	0	1	1	1	0
46,55	1	47	1	0	1	1	0	1
26,88	2	27	1	0	1	1	0	1
>60	0	>60	0	0	1	1	0	0
>60	0	>60	0	0	1	1	0	1
>60	0	>60	0	0	1	1	0	0
>60	0	>60	1	0	1	0	1	1
>60	0	>60	1	0	1	1	1	0
>60	0	>60	1	0	1	1	0	0
>60	0	>60	1	0	1	1	0	0
>60	0	>60	1	0	1	1	0	1
>60	0	>60	1	0	1	1	1	0
57,4	1	57	1	0	1	1	1	1
>60	0	>60	1	0	1	1	0	0
NA	0	NA	0	0	1	0	0	1
57,51	1	58	1	1	1	1	1	1
53	1	53	1	0	1	1	0	0
>60	0	>60	1	0	1	1	0	0
NA	0	NA	1	0	1	1	0	1
26,93	2	27	1	1	1	1	1	1
58,17	1	58	1	0	1	1	1	0
58	1	58	1	0	1	1	0	0
>60	0	>60	0	0	1	1	0	0
<60	0	<60	1	0	0	1	0	0
>60	0	>60	0	0	1	1	1	0
>60	0	>60	1	0	1	1	0	0
NA	0	NA	1	0	1	1	1	1
>60	0	>60	0	0	1	1	0	0
>60	0	>60	0	0	1	1	0	1
NA	0	NA	0	0	0	1	1	1
>60	0	>60	1	0	1	1	1	1
NA	0	NA	0	0	0	1	0	0
>60	0	>60	1	0	1	1	0	1
>60	0	>60	1	0	1	1	0	1
>60	0	>60	1	0	1	1	1	0
NA	0	NA	0	0	1	1	1	0
NA	0	NA	0	1	1	1	0	0

>60	0	>60	1	0	1	1	0	0
>60	0	>60	1	0	1	1	0	0
>60	0	>60	1	0	1	1	1	0
42,21	1	42	0	0	1	1	1	0
>60	0	>60	1	0	1	1	0	0
NA	0	NA	1	0	1	0	0	0
>60	0	>60	1	0	1	1	1	1
NA	0	NA	1	0	1	1	0	1
>60	0	>60	1	1	1	1	0	0
36,22	1	36	1	0	1	1	1	0
>60	0	>60	1	0	0	1	0	0
>60	0	>60	1	0	1	1	0	0
>60	0	>60	0	0	1	1	1	0
51,5	1	52	1	0	1	1	1	0
>60	0	>60	1	0	1	1	1	1
NA	0	NA	1	0	1	1	1	1
>60	0	>60	0	0	1	1	1	1
>60	0	>60	1	0	1	1	1	0
>60	0	>60	0	0	1	1	1	0
31,9	1	32	1	0	0	1	1	1
>60	0	>60	1	0	0	1	0	1
>60	0	>60	1	0	1	1	0	1
32	1	32	1	0	1	1	1	1
>60	0	>60	1	0	1	1	1	1
>60	0	>60	0	0	1	1	1	0
NA	0	NA	1	0	1	1	1	1
>60	0	>60	1	0	0	1	1	1
NA	0	NA	1	0	1	1	1	0
>60	0	>60	0	0	1	1	0	0
>60	0	>60	1	0	1	1	1	1
>60	0	>60	1	0	1	1	1	1
>60	0	>60	1	0	1	1	0	1
>60	0	>60	1	0	1	1	1	1
>60	0	>60	1	0	1	1	1	1
NA	0	NA	0	0	1	1	1	1
NA	0	NA	0	0	1	1	0	0
>60	0	>60	1	0	1	1	0	0
>60	0	>60	1	0	1	1	1	1
NA	0	NA	0	0	1	1	1	0
>60	0	>60	1	0	1	0	0	0
>60	0	>60	1	0	1	1	0	0
>60	0	>60	1	0	1	1	1	1
>60	0	>60	1	0	1	1	1	1
>60	0	>60	0	0	1	1	0	0
>60	0	>60	0	0	1	0	1	0
25,68	2	26	0	0	1	1	0	1
NA	0	NA	0	0	1	1	1	0
32	1	32	1	0	1	1	1	1
47	1	47	0	0	1	1	1	1
>60	0	>60	0	0	1	1	0	0

>60	0	>60	1	0	1	1	0	0
>60	0	>60	0	0	1	1	0	0
NA	0	NA	1	0	1	1	1	1
>60	0	>60	0	0	1	1	0	0
13,5	2	14	1	0	1	1	0	1
NA	0	NA	1	0	1	1	1	0
>60	0	>60	1	0	1	1	1	0
>60	0	>60	1	0	1	1	0	0
53,49	1	53	1	0	1	1	0	1
53,49	1	53	1	0	1	1	1	1
>60	0	>60	0	0	1	1	1	0
>60	0	>60	1	0	1	1	0	0
NA	0	NA	0	0	1	1	0	1
>60	0	>60	1	0	1	1	0	0
56,62	1	57	1	0	1	1	1	0
56,39	1	56	1	1	1	1	1	0
>60	0	>60	1	0	1	1	0	0
>60	0	>60	0	0	1	1	0	0
>60	0	>60	1	0	1	0	1	1
54	1	54	1	0	1	0	0	1
58,64	1	59	0	0	1	1	0	1
>60	0	>60	1	0	1	1	1	0
>60	0	>60	1	0	1	1	1	0
NA	0	NA	1	0	1	1	0	0
>60	0	>60	0	0	1	1	1	0
10	2	10	0	0	1	1	0	1
>60	0	>60	1	0	1	0	0	0
>60	0	>60	0	0	1	1	0	0
55,06	1	55	1	0	1	1	0	1
>60	0	>60	1	0	1	1	0	1
NA	0	NA	1	0	1	1	1	1
>60	0	>60	1	0	1	1	1	1
>60	0	>60	0	0	1	1	1	1
>60	0	>60	0	0	0	0	1	1
NA	0	NA	0	0	1	1	0	0
NA	0	NA	1	0	1	1	1	1
>60	0	>60	1	0	1	1	1	0
NA	0	NA	0	0	1	1	0	0
NA	0	NA	1	0	1	1	1	1
>60	0	>60	1	0	1	1	0	0
NA	0	NA	1	0	1	1	0	0
>60	0	>60	0	0	1	1	0	0
>60	0	>60	1	0	1	1	1	0
48,48	1	48	0	0	1	0	0	1

Statins	anti-platel	Smoking	CRT	ICD	QRS	QRS.Divisio	Hemoglobi	BNP
1	1	1	CRT-	ICD-	160	QRS>120	9,3	NA
1	1	1	CRT-	ICD-	160	QRS>120	13,5	226
1	1	1	CRT-	ICD+	240	QRS>120	14,4	80
1	1	1	CRT-	ICD+	120	QRS>120	14,6	75
1	1	1	CRT-	ICD-	120	QRS>120	12,6	NA
1	1	1	CRT-	ICD+	110	QRS<120	15,4	611
1	1	1	CRT-	ICD+	80	QRS<120	13,9	202
0	1	0	CRT-	ICD+	120	QRS>120	15,1	NA
1	0	1	CRT-	ICD+	90	QRS<120	NA	NA
1	1	1	CRT-	ICD+	120	QRS>120	16,7	80
0	1	1	CRT-	ICD-	80	QRS<120	14,1	NA
1	1	1	CRT-	ICD-	80	QRS<120	13,9	NA
1	1	1	CRT-	ICD+	160	QRS>120	13,2	4323
1	1	1	CRT-	ICD+	160	QRS>120	NA	NA
1	1	0	CRT-	ICD-	100	QRS<120	13,9	600
1	0	1	CRT-	ICD+	200	QRS>120	14,8	NA
1	1	1	CRT-	ICD-	95	QRS<120	13,8	35
1	1	1	CRT-	ICD+	130	QRS>120	11,5	161
1	1	1	CRT-	ICD+	90	QRS<120	14,7	80
1	1	1	CRT-	ICD+	130	QRS>120	15,5	100
1	1	1	CRT-	ICD-	80	QRS<120	14,3	85
1	1	1	CRT-	ICD+	200	QRS>120	15,1	448
1	1	1	CRT-	ICD+	80	QRS<120	14,8	NA
1	1	0	CRT-	ICD+	90	QRS<120	NA	NA
1	1	0	CRT-	ICD+	120	QRS>120	14,6	4323
1	1	1	CRT-	ICD-	100	QRS<120	9,6	4323
1	1	1	CRT-	ICD+	110	QRS<120	15,6	237
0	1	1	CRT-	ICD+	100	QRS<120	14,7	238
0	1	0	CRT-	ICD+	90	QRS<120	14,2	300
1	1	1	CRT-	ICD-	80	QRS<120	NA	NA
1	1	1	CRT-	ICD-	80	QRS<120	15,2	NA
1	1	1	CRT-	ICD+	170	QRS>120	12,3	393
0	1	1	CRT-	ICD+	100	QRS<120	15,6	NA
1	1	1	CRT-	ICD-	70	QRS<120	14,3	74
1	1	1	CRT-	ICD+	120	QRS>120	NA	NA
1	1	1	CRT+	ICD+	160	QRS>120	8,6	405
1	1	0	CRT-	ICD-	80	QRS<120	NA	NA
1	1	0	CRT-	ICD-	80	QRS<120	NA	NA
1	1	1	CRT-	ICD+	90	QRS<120	13,8	NA
1	1	1	CRT-	ICD-	80	QRS<120	NA	NA
1	1	1	CRT-	ICD-	80	QRS<120	15,1	NA
1	1	1	CRT-	ICD-	70	QRS<120	14,8	47
1	1	0	CRT-	ICD+	120	QRS>120	15,6	NA
1	1	1	CRT-	ICD+	90	QRS<120	13,6	62
1	1	1	CRT-	ICD+	120	QRS>120	13,9	1047
1	1	0	CRT-	ICD+	90	QRS<120	11,6	200
1	1	0	CRT-	ICD-	100	QRS<120	13,6	2305
1	1	1	CRT-	ICD+	140	QRS>120	14	NA
1	0	1	CRT-	ICD+	160	QRS>120	14,5	108

1	1	1	CRT-	ICD-	100	QRS>120	NA	497
1	0	0	CRT-	ICD-	160	QRS>120	NA	NA
1	1	0	CRT+	ICD+	160	QRS>120	8,9	NA
1	1	0	CRT-	ICD+	160	QRS>120	14,4	80
1	1	1	CRT-	ICD-	100	QRS<120	15,3	NA
1	1	1	CRT-	ICD+	80	QRS<120	12,5	50
1	1	1	CRT-	ICD+	120	QRS>120	14,6	NA
1	1	1	CRT+	ICD+	200	QRS>120	13,1	100
1	1	1	CRT-	ICD+	120	QRS>120	14,8	NA
1	1	1	CRT-	ICD-	100	QRS<120	14,6	14
0	1	1	CRT-	ICD+	100	QRS<120	NA	NA
1	1	1	CRT-	ICD-	70	QRS<120	10,5	165
1	1	1	CRT-	ICD+	100	QRS<120	14,1	77
1	1	1	CRT-	ICD-	80	QRS<120	12,8	87
1	1	0	CRT-	ICD+	90	QRS<120	13,1	1047
1	1	0	CRT+	ICD+	150	QRS>120	12,7	NA
1	1	0	CRT+	ICD+	150	QRS>120	13,8	NA
1	1	0	CRT-	ICD-	80	QRS<120	16,6	NA
1	1	1	CRT-	ICD+	120	QRS>120	NA	NA
1	1	1	CRT-	ICD+	120	QRS>120	12,3	336
1	1	1	CRT-	ICD-	90	QRS<120	14,7	66
1	1	1	CRT-	ICD-	90	QRS<120	NA	NA
1	1	1	CRT-	ICD-	90	QRS<120	13,8	NA
1	1	1	CRT-	ICD+	120	QRS>120	14,8	NA
1	1	1	CRT-	ICD-	100	QRS<120	NA	NA
1	1	0	CRT-	ICD+	160	QRS>120	13,4	3640
1	1	1	CRT-	ICD-	110	QRS<120	NA	NA
1	1	0	CRT-	ICD+	100	QRS<120	NA	NA
1	1	0	CRT-	ICD+	120	QRS>120	11,2	4443
0	1	1	CRT-	ICD+	80	QRS<120	NA	NA
1	1	1	CRT-	ICD-	200	QRS>120	NA	NA
1	1	1	CRT-	ICD+	120	QRS>120	15	NA
1	1	1	CRT-	ICD+	130	QRS>120	12,1	1045
1	1	1	CRT+	ICD+	140	QRS>120	14,4	NA
1	1	1	CRT-	ICD+	100	QRS<120	NA	NA
0	0	0	CRT-	ICD-	100	QRS<120	NA	NA
1	1	0	CRT+	ICD+	120	QRS>120	NA	NA
1	1	1	CRT-	ICD+	120	QRS>120	13,6	300
1	1	0	CRT-	ICD-	90	QRS<120	NA	NA
1	1	0	CRT-	ICD+	120	QRS>120	NA	200
1	1	0	CRT-	ICD+	110	QRS<120	12	NA
0	1	1	CRT-	ICD-	120	QRS>120	NA	1035
1	1	1	CRT-	ICD+	120	QRS>120	NA	NA
1	1	0	CRT-	ICD+	80	QRS<120	15,6	NA
1	1	1	CRT-	ICD+	90	QRS<120	12	120
1	1	1	CRT-	ICD+	120	QRS>120	14,5	67
1	1	1	CRT-	ICD-	160	QRS>120	15,7	300
1	1	1	CRT-	ICD+	120	QRS>120	11,5	68
1	1	0	CRT-	ICD-	80	QRS<120	NA	NA
0	0	1	CRT-	ICD+	100	QRS<120	11,7	200

1	0	0	CRT-	ICD-	60	QRS<120	14,3	27
1	1	1	CRT-	ICD+	110	QRS<120	NA	NA
1	1	1	CRT-	ICD+	120	QRS>120	14,5	64
1	1	1	CRT-	ICD+	160	QRS>120	13,7	104
1	1	0	CRT-	ICD+	110	QRS<120	12,5	83
1	1	1	CRT-	ICD-	120	QRS>120	NA	130
1	1	1	CRT-	ICD-	140	QRS>120	NA	NA
1	1	1	CRT-	ICD-	150	QRS>120	NA	102
1	1	1	CRT-	ICD+	NA	QRS<120	14	NA
1	1	1	CRT-	ICD+	120	QRS>120	NA	NA
1	1	0	CRT-	ICD-	140	QRS>120	14,1	112
1	1	1	CRT-	ICD-	115	QRS<120	14,8	156
1	1	0	CRT-	ICD-	140	QRS>120	NA	NA
1	1	1	CRT-	ICD-	160	QRS>120	13	NA
1	1	1	CRT+	ICD+	200	QRS>120	NA	NA
1	1	1	CRT-	ICD-	120	QRS>120	13,7	104
1	1	1	CRT-	ICD+	110	QRS<120	10,5	2330
1	1	1	CRT-	ICD-	110	QRS<120	12,2	21
0	0	0	CRT-	ICD+	100	QRS<120	NA	NA
1	0	1	CRT-	ICD+	160	QRS>120	14,5	64
1	1	1	CRT-	ICD+	110	QRS<120	NA	NA
1	1	0	CRT-	ICD-	80	QRS<120	11,9	NA
1	1	1	CRT-	ICD-	120	QRS>120	13,7	104
1	0	0	CRT-	ICD-	115	QRS<120	14	64
0	0	1	CRT-	ICD-	100	QRS<120	15,3	33
1	1	1	CRT-	ICD+	80	QRS<120	NA	NA
1	1	1	CRT+	ICD+	170	QRS>120	NA	NA
1	1	1	CRT-	ICD-	80	QRS<120	14,1	62
1	1	1	CRT-	ICD-	100	QRS<120	15,6	NA
1	1	1	CRT-	ICD+	120	QRS>120	NA	NA
1	0	1	CRT-	ICD+	120	QRS>120	13,8	106
1	1	1	CRT-	ICD+	90	QRS<120	16,8	NA
1	1	1	CRT-	ICD+	100	QRS<120	14,8	NA
1	1	1	CRT+	ICD+	200	QRS>120	13,7	104
1	1	0	CRT-	ICD-	180	QRS>120	NA	NA
1	0	1	CRT-	ICD-	80	QRS<120	15,2	126
1	1	1	CRT-	ICD-	90	QRS<120	14,8	105
1	1	1	CRT-	ICD+	120	QRS>120	14,5	64
1	1	1	CRT-	ICD-	120	QRS>120	13,7	104
0	1	1	CRT-	ICD-	200	QRS>120	NA	NA
1	1	1	CRT-	ICD-	120	QRS>120	NA	NA
1	1	1	CRT-	ICD+	120	QRS>120	14,2	1680
1	1	0	CRT+	ICD+	180	QRS>120	15,7	NA
1	1	1	CRT-	ICD+	80	QRS<120	13,8	106
1	1	1	CRT-	ICD-	100	QRS<120	13,8	223
1	0	1	CRT-	ICD+	NA	QRS<120	16,8	NA
1	1	1	CRT-	ICD-	100	QRS<120	11,7	NA
1	1	0	CRT-	ICD+	110	QRS<120	NA	NA
1	1	0	CRT-	ICD+	110	QRS<120	11,6	50
1	1	1	CRT-	ICD-	80	QRS<120	NA	NA

1	1	1	CRT-	ICD-	80	QRS<120	12,7	213
1	1	1	CRT-	ICD+	180	QRS>120	14,5	64
1	1	1	CRT-	ICD-	120	QRS>120	13,7	104
1	1	1	CRT+	ICD+	200	QRS>120	NA	NA
1	1	1	CRT-	ICD+	200	QRS>120	14,4	NA
1	1	1	CRT-	ICD-	100	QRS<120	NA	NA
1	0	1	CRT-	ICD-	100	QRS<120	14,9	36
0	1	0	CRT-	ICD-	100	QRS<120	NA	NA
1	0	1	CRT-	ICD+	120	QRS>120	16,8	NA
1	1	0	CRT+	ICD+	160	QRS>120	NA	NA
1	1	1	CRT+	ICD+	200	QRS>120	NA	NA
1	1	1	CRT-	ICD+	100	QRS<120	14,8	NA
0	1	0	CRT-	ICD-	80	QRS<120	NA	NA
1	1	0	CRT-	ICD+	80	QRS<120	13,7	104
1	1	1	CRT-	ICD+	80	QRS<120	14,4	NA
1	1	1	CRT+	ICD+	100	QRS<120	NA	NA
1	1	1	CRT-	ICD+	100	QRS<120	13,8	NA
0	1	1	CRT-	ICD-	80	QRS<120	14,8	NA
1	1	1	CRT-	ICD-	100	QRS<120	12,3	NA
1	1	1	CRT-	ICD-	100	QRS<120	NA	NA
1	1	1	CRT-	ICD+	160	QRS>120	12	NA
1	1	1	CRT-	ICD+	130	QRS>120	NA	NA
1	1	0	CRT-	ICD+	125	QRS>120	14,4	NA
1	1	0	CRT-	ICD+	130	QRS>120	NA	NA
1	1	1	CRT-	ICD-	160	QRS>120	13,8	157
1	1	0	CRT+	ICD+	110	QRS<120	12	800
1	1	1	CRT-	ICD-	110	QRS<120	11,4	759
1	1	0	CRT-	ICD+	100	QRS<120	NA	NA
1	1	1	CRT-	ICD+	120	QRS>120	16,1	80
1	1	1	CRT-	ICD+	160	QRS>120	14,1	340
1	1	0	CRT-	ICD+	110	QRS<120	14,4	200
0	1	0	CRT-	ICD-	120	QRS>120	NA	NA
1	1	1	CRT-	ICD+	130	QRS>120	12,9	130
1	1	1	CRT-	ICD-	110	QRS<120	14,5	61
1	1	1	CRT-	ICD+	130	QRS>120	13,3	100
0	0	0	CRT-	ICD+	120	QRS>120	14	NA
1	1	1	CRT-	ICD-	80	QRS<120	14	53
1	1	1	CRT-	ICD+	80	QRS<120	NA	NA
1	1	1	CRT-	ICD+	160	QRS>120	13,9	NA
1	1	0	CRT-	ICD-	110	QRS<120	NA	NA
1	1	1	CRT-	ICD-	110	QRS<120	NA	NA
1	1	1	CRT-	ICD-	100	QRS<120	14,1	215
0	0	1	CRT+	ICD+	160	QRS>120	NA	NA
1	1	0	CRT-	ICD+	160	QRS>120	14,3	801

LADs	EDV	ESV	GLS
48	140	69	-15,1
50	118	68	-8,3
51	119	68	-7,3
51	156	100	NA
54	170	135	-15,1
48	171	87	NA
43	165	88	-15,2
50	155	67	-15,6
NA	187	89	-16,6
38	135	78	NA
41	79	48	NA
45	94	58	-14,9
55	95	67	-14,2
NA	101	56	-14,7
45	183	78	NA
50	195	120	-5,6
35	133	66	-12,5
48	100	67	-13,4
48	165	78	-12,1
55	98	78	NA
47	157	84	-16,6
50	88	67	-17,6
42	150	95	-15,7
48	170	107	-4,3
50	172	77	NA
43	144	66	-16,7
42	132	66	NA
41	176	73	-7,2
42	115	78	-13,6
46	117	77	-17,4
40	113	78	NA
46	286	211	-18,4
44	199	148	-12,7
42	95	58	-12,1
NA	178	130	-12,4
32	95	70	NA
NA	88	67	NA
52	98	54	-11
48	98	67	-12,1
NA	99	66	-12
38	120	60	-10
41	147	88	-12,6
48	127	87	-12,5
33	112	63	-13,3
51	268	206	NA
47	266	200	-14,1
43	256	189	-3,4
43	164	104	-16,7
52	114	77	-5,6

	46	207	126	-8
	47	160	100	-7,8
	51	256	181	-9,3
	39	NA	NA	-12,3
	41	115	78	-9,2
	43	220	102	-10,2
	50	118	78	NA
	38	133	96	NA
	49	220	160	NA
	36	125	78	-14,1
NA		116	67	NA
	40	106	55	-12,1
	40	NA	NA	NA
	39	165	88	-10,8
	49	266	210	-7,8
	39	76	57	-6,5
	46	208	154	NA
	49	200	115	NA
	54	200	130	-14,2
	27	92	76	-9,5
	37	135	68	-15,3
	36	197	100	-15,4
	40	159	82	-18,3
	54	134	98	-19,2
	39	149	96	-7,8
	48	146	76	-8,9
	39	188	121	-7,7
	47	177	100	NA
	58	318	258	NA
	43	188	88	-13,5
	56	217	155	-14,2
	58	89	56	-15,1
	45	187	98	-6,5
	56	196	113	-4,8
NA		194	87	NA
	30	155	99	-9,9
NA		140	90	-7,9
	51	160	125	-15,6
	38	125	78	-10,2
	49	209	170	NA
	38	78	56	-13,9
	47	208	134	-13,7
	54	115	67	NA
NA		176	90	-12,6
	45	118	78	NA
	45	198	99	-6,5
	36	98	56	-17,6
	48	109	71	-15,4
NA		121	67	NA
	60	100	67	-6,2

40	95	42	-16,8
NA	94	56	-16,7
44	97	54	NA
41	135	56	NA
54	220	140	-12,1
45	78	55	-12,3
47	211	143	-8,4
NA	273	173	-13,4
45	229	146	NA
44	136	91	-8,6
49	84	49	-9,8
63	133	77	-12,8
29	167	101	-16
47	191	114	-12,1
40	259	206	-4,9
41	110	78	-10
63	126	85	NA
32	128	74	-15
55	112	66	-15
45	119	81	NA
45	139	107	-6,9
42	74	38	-15,1
41	114	68	NA
47	166	84	-15,3
44	198	129	-10,9
41	NA	NA	-7,1
48	205	170	-8,7
NA	84	52	-13,1
44	157	90	-14,3
42	191	128	-9,7
39	130	90	-8,8
34	NA	NA	-5,4
39	116	78	-10
41	135	67	NA
43	118	89	NA
49	94	55	-16,7
42	157	86	-14
44	155	100	-14,1
41	155	101	NA
42	160	100	-15,6
NA	154	98	-15,6
45	210	171	-5,5
45	330	240	NA
45	118	78	-13,4
35	102	55	-11,8
50	153	113	-3,4
60	114	67	-12,5
NA	122	66	-16,7
34	115	69	-8,2
NA	117	78	NA

45	182	118	-9,8
50	270	215	-10,3
41	156	NA	-11,2
44	256	210	-11,4
46	145	NA	-14
47	264	163	-11,2
46	184	111	-18
NA	144	100	NA
34	NA	NA	-12,1
38	117	97	-5,2
NA	NA	NA	-9,9
39	116	66	-10,2
45	160	83	-14,9
41	NA	NA	-15,1
46	155	67	-8,7
42	173	150	-6,9
45	143	89	-13,4
45	142	77	-16,1
NA	141	77	-17,2
42	146	104	-4,8
43	323	273	-6,6
49	141	107	-15
NA	140	88	-14,2
39	140	98	NA
45	158	93	-13,8
48	322	187	NA
52	140	77	-14,5
37	157	87	-15,1
46	289	188	NA
43	298	177	-6,5
46	156	90	-16,1
53	267	181	-12,3
40	177	115	NA
33	198	134	-12,6
42	82	51	NA
45	NA	NA	-19,1
42	107	55	-17,8
NA	NA	NA	-19,1
49	350	290	-7,2
NA	NA	NA	-18,2
40	227	130	-17,4
45	243	133	-10,1
30	106	71	-9,2
50	128	98	-9,8
